# Extrinsic MAVS signaling is critical for Treg maintenance of Foxp3 expression following acute flavivirus infection

**DOI:** 10.1038/srep40720

**Published:** 2017-01-17

**Authors:** Andreia Da Costa, Esteban Garza, Jessica B. Graham, Jessica L. Swarts, Andrew G. Soerens, Michael Gale, Jennifer M. Lund

**Affiliations:** 1 Vaccine and Infectious Disease Division, Fred Hutchinson Cancer Research Center, Seattle, WA, USA; 2 Graduate Program in Pathobiology, Department of Global Health, University of Washington, Seattle, WA, USA; 3 Center for Innate Immunity and Immune Disease, Department of Immunology, University of Washington School of Medicine, Seattle, WA, USA

## Abstract

Given the rapid spread of flaviviruses such as West Nile virus (WNV) and Zika virus, it is critical that we develop a complete understanding of the key mediators of an effective anti-viral response. We previously demonstrated that WNV infection of mice deficient in mitochondrial antiviral-signaling protein (MAVS), the signaling adaptor for RNA helicases such as RIG-I, resulted in increased death and dysregulated immunity, which correlated with a failure of Treg expansion following infection. Thus, we sought to determine if intrinsic MAVS signaling is required for participation of Tregs in anti-WNV immunity. Despite evidence of increased Treg cell division, Foxp3 expression was not stably maintained after WNV infection in MAVS-deficient mice. However, intrinsic MAVS signaling was dispensable for Treg proliferation and suppressive capacity. Further, we observed generation of an effective anti-WNV immune response when Tregs lacked MAVS, thereby demonstrating that Treg detection of the presence of WNV through the MAVS signaling pathway is not required for generation of effective immunity. Together, these data suggest that while MAVS signaling has a considerable impact on Treg identity, this effect is not mediated by intrinsic MAVS signaling but rather is likely an effect of the overproduction of pro-inflammatory cytokines generated in MAVS-deficient mice after WNV infection.

The immune response is tightly regulated to maximize potential anti-microbial responses such that infections can be cleared, leaving behind life-long, protective memory responses, while at the same time sparing host tissues and preventing extensive autoimmunity and immunopathology. One method by which the immune system achieves this balance is through self/non-self discrimination, or recognition of pathogen-associated molecular patterns (PAMPs) through pattern recognition receptors (PRRs). While this level of recognition has traditionally been characterized within antigen-presenting cells (APCs), recent studies have examined the potential for T cells to sense the presence of foreign products through PRRs^1^. In particular, T cells express various TLRs, and T cells were first reported to respond to direct TLR stimulation when it was observed that in an APC-free cell culture, TCR-triggered T cells could be further stimulated to produce IL-2 and to proliferate by the TLR9 agonist CpG^2^. Since that initial finding, CD4+ and CD8+ T cell stimulation through various TLRs has been shown to promote antigen-specific proliferation, cell survival, homeostatic proliferation of memory cells, and cytokine expression^1^,^3^,^4^,^5^,^6^,^7^,^8^. In addition, regulatory T cells (Treg), a subset of CD4+ T cells expressing the transcription factor Foxp3, were shown to express TLR8, ligation of which inhibited their suppressive function in humans^9^. Conversely, it has been reported that LPS stimulation of Tregs induces proliferation and enhanced suppressive capacity in the murine system^10^.

A second class of PRRs, the RIG-I-like receptor (RLR) family, has also been directly implicated in T cell responses. RIG-I is a cytoplasmic RNA sensor that signals the activation of interferon regulatory factor (IRF)-3 and induction of Type I interferon production to initiate the innate immune response to infection. RIG-I signaling is mediated by its downstream obligate adaptor protein, MAVS. T cells, including Tregs, express RIG-I^11^,^12^ indicating that this protein could have a role in intracellular RNA detection. Further, stimulation of Tregs with Encephalomyocarditis virus has been shown to decrease Treg inhibitory function in an MDA5-dependent manner^11^. Indeed, a recent study examining the importance of MAVS in immune control of West Nile virus (WNV) infection demonstrated that mice lacking this protein have a phenotype similar to mice depleted of the Treg subset^13^. Specifically, WNV-infected MAVS knockout mice displayed uncontrolled inflammation, increased pro-inflammatory cytokine and chemokine production, increased numbers of DCs, and increased virus-specific T cell responses, all correlated with a lack of Treg expansion following infection^13^, similar to what was observed in mice conditionally depleted of Tregs prior to virus infection with HSV-2 and WNV^14^,^15^,^16^. Given the demonstrated role of Tregs in immunity to various pathogens, performing such functions as limiting protective anti-viral immune responses as well as limiting immune-mediated inflammation and tissue destruction^17^ and promoting proper CD4 T cell priming^18^, enhancing our understanding of how these cells detect and respond to infection may be critical to designing therapeutic strategies to limit and cure infectious diseases.

We hypothesized that Tregs may directly detect infection by WNV through the RLR signaling pathway with strict dependence on the MAVS adaptor protein for downstream signaling, thereby altering the suppressive capacity of Tregs and the ensuing anti-viral immune response. To test this hypothesis, we used a congenically marked mixed bone marrow chimera mouse model. Briefly, mice expressing the human diphtheria toxin receptor (DTR) inserted into the Foxp3 locus (Foxp3^*DTR*^ mice)^19^ were irradiated prior to intravenous transfer of donor bone marrow containing a 90% Foxp3^*DTR*^/10% Mavs^−/−^ or 90% Foxp3^*DTR*^/10% WT bone marrow mixture. Following a period of recovery and reconstitution, chimeras were depleted of all DTR-bearing Tregs via administration of diphtheria toxin, thereby allowing for WT or Mavs^−/−^ donor Tregs to expand from 10% of the Treg population to >90% of total Tregs. Employing this strategy allowed us to test the requirement for direct MAVS signaling in Tregs in the immune response to WNV. We conclude that MAVS is not essential for Treg response to WNV, indicating that sensing of WNV is likely mediated by other processes such as inflammatory cytokine signals.

## Results

### Foxp3 expression is down-regulated in Tregs following WNV infection of Mavs^−/−^ mice

**Figure 1:**
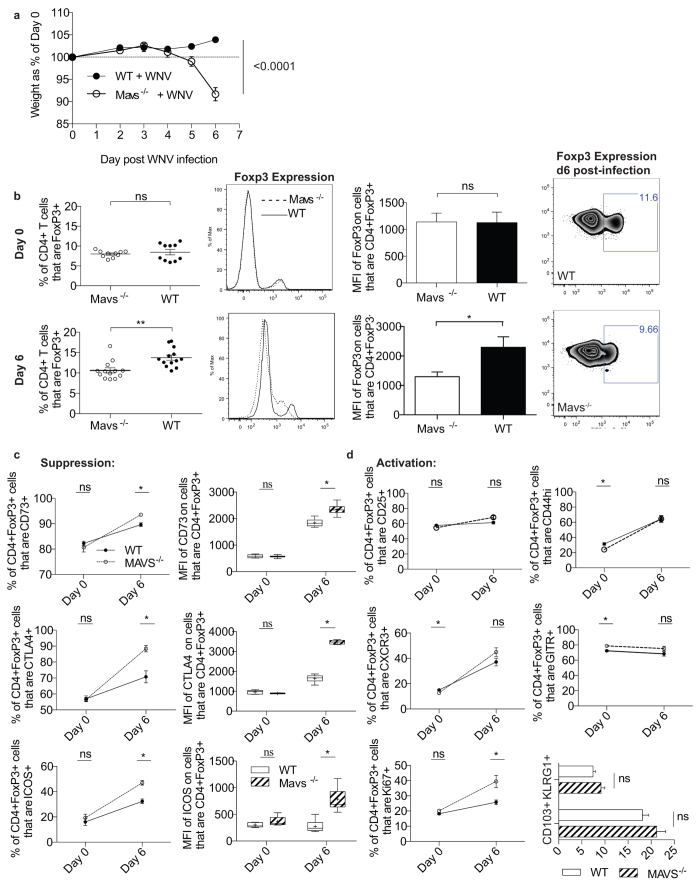
Foxp3 expression is down-regulated in Mavs^−/−^ mice after WNV infection. (**a**) Weight loss of WT and Mavs^−/−^ mice was assessed following s.c. footpad infection with 1000 PFU WNV. (**b**) Foxp3 expression in splenic lymphocytes was assessed through flow cytometry in both naïve (Day 0) and WNV infected (Day 6) Mavs^−/−^ mice and compared to age matched WT controls. After per cell analysis of Foxp3 expression through median fluorescence intensity (MFI), we find that the Treg frequency difference is due to down-regulation of Foxp3 expression. (**c**) Treg suppressive molecule expression was examined by flow cytometry following 6d of WNV infection. (**d**) Activation of Tregs after infection in KO and WT controls. Data represents 2 independent experiments (D0 n = 10 WT/KO), (D6 n = 13 WT/KO). Statistical significance calculated using two-tailed unpaired Student’s *t* tests, with * indicating p < 05. Error bars reflect +/− SEM. Whisker box plots display min and max values; +indicates mean value.

Our previous work suggested that Tregs in Mavs^−/−^ mice failed to proliferate in response to WNV infection as compared to WT controls^13^. Therefore, we further investigated this finding in order to fully characterize the Treg response in the complete absence of MAVS signaling. In particular, we examined the frequency and phenotype of Tregs within Mavs^−/−^ mice during WNV infection to determine if the expression of key suppressive molecules was altered in this system. We found that in addition to the previously described secondary lymphoid organ enlargement^13^, WNV-infected Mavs^−/−^ mice rapidly declined beginning at day 5 p.i., exhibiting dramatic weight loss (Fig. 1a) and symptoms such as ruffled fur, lethargy and diarrhea. On day 6 p.i., we noted inflammation in intestinal tissues, discoloration in the liver, blackened gall bladders and enlarged mesenteric lymph nodes in Mavs^−/−^ but not MAVS-sufficient groups (data not shown). These symptoms indicate that WNV infection in Mavs^−/−^ mice leads to the development of a grossly dysregulated systemic inflammatory response with gut involvement similar to that seen in DSS colitis models in Mavs^−/−^ mice^20^. When we examined the frequency of CD4+Foxp3+ Tregs, we found that in contrast to pre-infection, Treg frequency was significantly reduced in Mavs^−/−^ mice after WNV infection as compared to WT controls. However, upon per cell analysis of Foxp3 expression through median fluorescence intensity (MFI) measurement, we found reduced Foxp3 fluorescence (Fig. 1b). The degree of Foxp3 expression has been shown to be directly associated with Treg suppressive capability in both Foxp3 attenuation models^21^ and *ex vivo* allograft transplantation models^22^, where decreased expression leads to the development of scurfy-like autoimmunity. Thus, we assessed the frequency of Treg-associated suppressive markers, and unexpectedly found that Mavs^−/−^ Tregs have a statistically significant increase in expression of all suppressive molecules measured, including CD73, CTLA-4, and ICOS (Fig. 1c). This paradoxical reduction in Foxp3 expression and concomitant alteration of suppressive profile may be a reflection of enrichment of less plastic Tregs within the heterogeneous Treg population represented in the spleen^23^. In addition, the extreme pro-inflammatory environment that develops in the Mavs^−/−^ mice after WNV could be contributing to a loss of Treg identity where a subset of Tregs has lost the ability to express Foxp3 entirely and/or the per cell expression of Foxp3 has dimmed sufficiently that it is no longer discernible. To further characterize Mavs^−/−^ Tregs in infected mice, we then examined the activation phenotype of these cells and found that the expression of CD44 and CXCR3 was increased after WNV infection as expected (Fig. 1d). However, despite the inflammatory conditions in the infected KO mice, Mavs^−/−^ Treg expression of CD44, CD25, and CXCR3 was comparable to WT counterparts. To determine Treg functional potential, we assessed the frequency of KLRG1 and CD103, proteins recently associated with terminal differentiation in Tregs^24^ (Fig. 1d), and found that they were similar regardless of MAVS expression. Finally, we found that the cell-cycle progression intracellular protein, Ki67, was significantly increased in Mavs^−/−^ Tregs, further suggesting that the decreased Treg frequency measured in Mavs^−/−^ mice after WNV infection is more likely due to reduced Foxp3 expression than a failure of Treg division.

**Figure 2:**
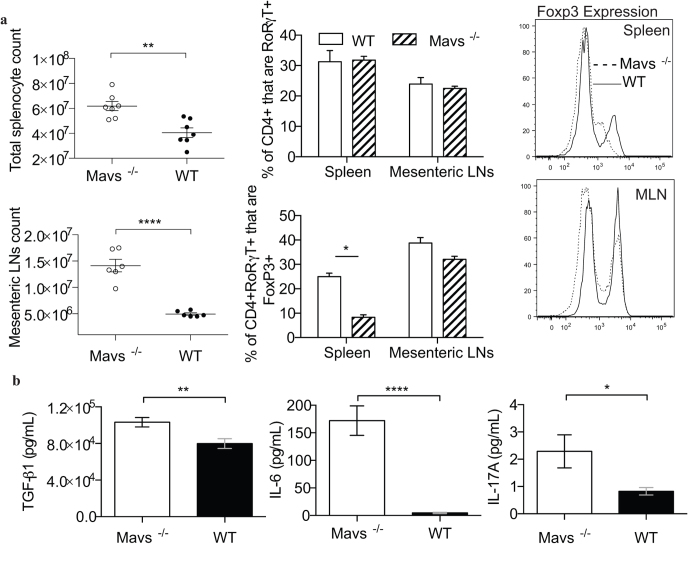
Foxp3 down regulation occurs in the presence of Th17 polarizing conditions. (**a**) Flow cytometric assessment of WT and Mavs^−/−^ spleen and mesenteric LN cells at d6 p.i. with WNV, as in Fig. 1. (**b**) IL-6, TGFβ and IL-17 levels were measured by ELISA in serum samples taken d6 post-WNV infection. 2B represents two independent experiments total n = 23 WT/Mavs^−/−^. Statistical significance was calculated using two-tailed unpaired Student’s *t* tests. Error bars reflect +/− SEM.

Since Tregs in Mavs^−/−^ mice appeared to be losing expression of Foxp3 following infection with WNV, along with experiencing diarrhea and other signs of gastrointestinal distress, we extended our analysis of Tregs to include the mesenteric lymph nodes (MLN). Mavs^−/−^ mice experienced not only profound splenomegaly, but also a significant enlargement of the MLN at day 6 p.i. (Fig. 2a). As an inappropriately regulated Th17 response has been previously implicated in gastrointestinal inflammation, we examined the frequency of RORγT+ CD4 T cells in the spleen and MLN, and while they did not vary significantly regardless of MAVS expression, there were significantly fewer RORγT+ CD4 T cells expressing Foxp3, based on both frequency and MFI, in the absence of MAVS (Fig. 2a). Similarly, Tregs have been observed to convert from suppressive cells to inflammatory Th17 cells upon treatment with polarizing extrinsic cytokine signals^25^,^26^. In support of the notion that Tregs require MAVS to maintain Foxp3 expression and thereby stable lineage commitment following WNV infection, Mavs^−/−^ mice had significantly elevated serum TGFβ and IL-6 levels following WNV infection (Fig. 2b). In combination TGFβ and IL-6 are known to support conversion of Tregs to Th17 cells^25^,^26^, and correspondingly Mavs^−/−^ mice had higher serum IL-17 (Fig. 2b). These results suggest that MAVS is required for Treg stability following WNV infection, though whether or not MAVS is required intrinsically remains uncertain.

### Intrinsic MAVS signaling is not required for Treg proliferation or suppressive function *in vitro*

**Figure 3:**
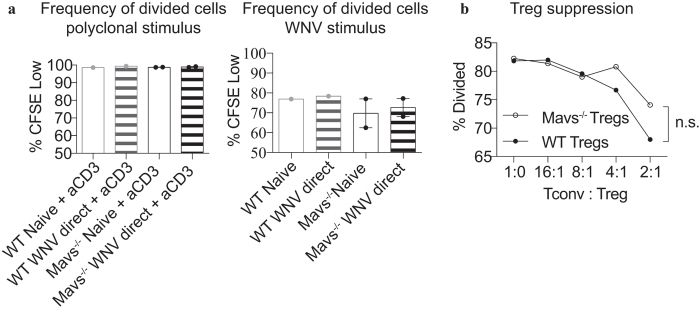
Instrinsic MAVS signaling is not required for Treg proliferation or suppressive function *in vitro*. (**a**) Tregs from either Mavs^−/−^ mice or WT mice were column-enriched based on CD4+CD25+ expression and stimulated as noted (left) or co-cultured with CD11c+ column enriched DCs (right). Samples were left either naïve or co-cultured with heat-inactivated whole WNV for 3 days. Anti-CD3 and anti-CD28 antibodies were used for polyclonal stimulation in positive control groups. Error bars reflect +/− SEM. (**b**) Spleens from Mavs^−/−^ or WT mice were harvested and homogenized. Tregs were column-enriched based on CD4+CD25+ expression. CD4+ (Tconv) cells were column-enriched through negative selection and CFSE labeled to track proliferation. Cells were co-cultured in decreasing Tconv: Treg proportions with irradiated splenocytes to serve as APCs and incubated for 80 hrs. Statistical significance calculated using two-tailed unpaired Student’s *t* tests.

Since our results demonstrated that WNV infection of Mavs^−/−^ mice led to decreased expression of Foxp3 compared to WT mice (Fig. 1b), likely accounting for the deficit of Treg expansion following infection with WNV (Fig. 1b)^13^, we next tested the role of intrinsic MAVS signaling in Treg proliferation *in vitro* using a standard CFSE proliferation assay. Initially, we tested the ability of Tregs to proliferate to a polyclonal TCR stimulus using a crosslinking antibody specific to CD3 and found that Mavs^−/−^ Tregs proliferated as well as WT Tregs. Further, as the frequency of Tregs that proliferated during the period of culture was extremely high, the addition of WNV to the culture was unable to augment proliferation and was again similar regardless of the presence of intrinsic MAVS in Tregs (Fig. 3a). In order to test the requirement for MAVS in Treg proliferation in response to WNV directly, we cultured Tregs with WNV and WT dendritic cells (DCs) without the addition of anti-CD3. WT and MAVS-deficient Tregs were equally able to proliferate under these conditions (Fig. 3a), suggesting that MAVS is dispensable for Treg proliferation upon TCR stimulus as well as in culture with WT DCs with or without WNV.

Although intrinsic MAVS was not required for Treg proliferation *in vitro*, it remained possible that intrinsic RLR signaling is required for Treg suppressive function, as Mavs^−/−^ mice suffered from several hallmarks of mice lacking functional Tregs, including a generally dysregulated immune response to WNV, enhanced numbers of immune effector cells present in the spleen, and profound splenomegaly^13^. Therefore, we tested the ability of Tregs lacking MAVS to suppress proliferation of conventional T cells using a CFSE-based suppression assay. Following three days of co-culture, WT and Mavs^−/−^ Tregs were equally proficient in suppressing T cell proliferation (Fig. 3b), suggesting that intrinsic MAVS signaling is not required for Treg suppressive function.

### Treg expression of MAVS does not affect WNV disease *in vivo*

**Figure 4:**
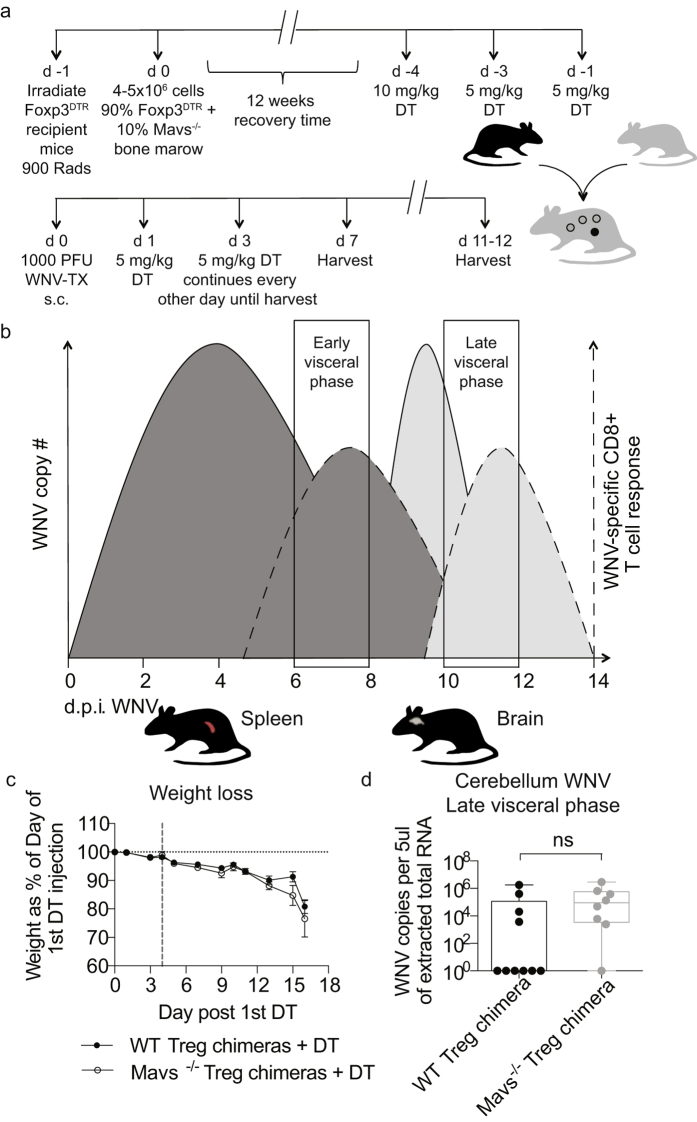
Treg-intrinsic MAVS does not play a role in disease outcome to WNV *in vivo*. (**a**) Schematic shows BM chimera generation and diphtheria toxin (DT) and WNV infection schedule. One day after irradiation, congenically marked Foxp3^DTR^ recipient mice received 90% Foxp3^DTR^ + 10% Mavs^−/−^ or WT bone marrow cells and allowed to recover for 12 weeks. DT was administered every other day until day 4 in order to ablate DTR expressing Tregs and allow Mavs^−/−^ or WT Tregs to expand and fill the niche. Mixed bone marrow chimeric mice were then infected s.c. with 1000 PFU WNV in the footpad to mimic natural infection. Mice were monitored for morbidity and mortality for up to 12 days post infection and then spleens and brains harvested. Tissues were analyzed for effector cell frequencies, counts and phenotype using flow cytometry. (**b**) WNV pathogenesis has been described as proceeding through three distinct phases: the Early phase during which WNV replicates within primarily infected skin tissue and migrates to the draining lymph nodes, the Visceral-organ dissemination phase (VOD phase) and the CNS phase^45^. Our work focuses on the resolution of the VOD phase and the potential impact of MAVS signaling in Tregs during this phase, by examining early VOD and late VOD events in WNV pathogenesis in subcutaneously infected mice. (**c**) Mice were monitored for weight loss through the infection course. Vertical dashed line indicates date of infection. There were no statistically significant differences found between groups. Statistical significance calculated using two-tailed unpaired Student’s *t* tests. Error bars reflect +/− SEM. (**d**) WNV infected mice were perfused with PBS and cerebellums collected from the brains of WT (n = 10) and Mavs^−/−^ (n = 8) chimeras on day 11–12 post infection. RNA was extracted and quantified using qRT-rtPCR. Whisker plots show min and max values. There were no statistically significant differences found between both groups. Statistical significance was calculated using two-tailed unpaired Student’s *t* tests.

Although *in vitro* data did not suggest a role for MAVS signaling in Tregs, given the *in vivo* alteration of Foxp3 expression in Mavs^−/−^ mice we further tested the role for this signaling molecule specifically in Tregs in the course of *in vivo* WNV infection. To test the role of MAVS exclusively in Tregs *in vivo*, we used an asymmetric mixed bone marrow chimera mouse model (Fig. 4a). Briefly, congenically labeled Foxp3^*DTR*^ mice were lethally irradiated and then infused with a 90% Foxp3^*DTR*^ Ly5.1 + 10% Mavs^−/−^ Ly5.2 (or WT Ly5.2) bone marrow mixture. By utilizing a 90/10 ratio, we were able to maximize the amount of hematopoietic stem cell precursors available for development of Tregs while simultaneously minimizing the contribution of other Mavs^−/−^ or WT cell types that might arise. Following a 3-month recovery time, diphtheria toxin (DT) was administered to deplete all DTR-bearing Tregs, thereby allowing Mavs^−/−^ or WT Tregs to expand to fill the compartment in response. Chimeras were then infected subcutaneously in the footpad to mimic the route of vector transmission of WNV. In particular, we focused on the visceral phase of WNV infection which can be split into an early phase, at approximately d7 p.i., and a late phase, at approximately d11–12 p.i., in which viral replication begins to wane in secondary lymphoid tissues and has transitioned across the blood brain barrier (Fig. 4b). After infection with WNV, mice were monitored daily for weight loss and clinical manifestations of disease, and mice with Mavs^−/−^ Tregs suffered from a nearly identical weight loss compared to mice with WT Tregs (Fig. 4c), suggesting that the course of disease was not altered when Tregs lacked MAVS. Additionally, we compared the viral burden within the CNS between Mavs^−/−^ Treg chimeric mice and WT controls. Real-time PCR on cerebellar tissues harvested during the late visceral phase revealed that there was no significant difference in WNV copy number between groups (Fig. 4d). Therefore, we conclude that Treg-intrinsic MAVS does not play a role in disease outcome to WNV *in vivo*.

### Intrinsic MAVS signaling is dispensable for Treg expansion and function following WNV infection *in vivo*

**Figure 5:**
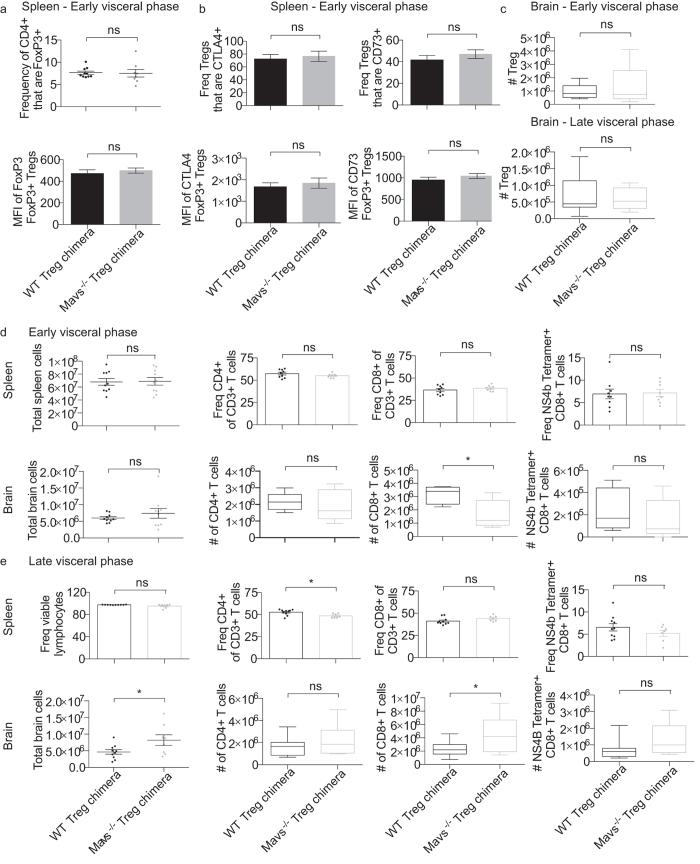
Intrinsic MAVS signaling is dispensable for Treg expansion and function following WNV infection *in vivo*. Mixed bone marrow chimeras were infected as in Fig. 4. (**a,b**) Expansion of donor BM-derived Foxp3+ Tregs in WT and Mavs^−/−^ chimeras. Bar graphs represent mean values of markers associated with Treg suppressive function. Error bars reflect +/− SEM. Statistical significance was calculated using two-tailed unpaired Student’s *t* tests. (**c–e**) Brains and spleens were harvested during early or late visceral phase (as described in Fig. 4b) post infection and analyzed via flow cytometry. (**c**) Number of BM-derived Foxp3+ Tregs in WT and Mavs^−/−^ chimeras in the brain in the early and late visceral phases. Early visceral phase (**d**) and late visceral phase (**e**) total cell counts and frequencies of CD4+ T cells, Tregs, CD8+ T cells, and WNV-specific CD8+ T cells in both spleen and brain following infection are shown. Brain whisker and box plots represent max. and min. values and are representative of 2 independent experiments. Statistical significance was calculated using two-tailed unpaired Student’s *t* tests. Error bars reflect +/− SEM.

Although we did not observe the increased viral burden and pathogenesis when only Tregs lacked MAVS as compared to the full knockout mouse^13^, it was possible that MAVS signaling in Tregs was required for a subtle element of the immune response to WNV. Therefore, we next examined the extent of Treg expansion following WNV infection. The frequency of Tregs present in the spleen was similar regardless of MAVS expression at the peak of the T cell response (Fig. 5a). In addition, we characterized the Treg response during infection by analyzing the frequency of a number of functional and migratory markers associated with suppressive function. CTLA-4 expression by Tregs was compared, as this is a key strategy used by Tregs to directly suppress antigen presenting cells and therefore the initiation of an immune response. However, splenic Tregs expressed similar levels of CTLA-4 regardless of MAVS expression (Fig. 5b). Tregs also employ a variety of other suppressive mechanisms including cAMP-mediated inhibition of surrounding effector cells^27^. We found that proteins critical to the function of Tregs such as CD73 and GITR (data not shown) were represented at similar frequencies despite a lack of MAVS expression in Tregs during infection. Furthermore, through median fluorescence intensity (MFI) analysis we found comparable expression of these molecules on a per-cell basis (Fig. 5b). Importantly, we found comparable levels of Foxp3 expression between Mavs^−/−^ Treg chimeras and WT controls (Fig. 5a), suggesting that instrinsic MAVS signaling is not required for stable expression of Foxp3 by Tregs.

In addition to playing a role in the secondary lymphoid organs, Tregs play a role in the central nervous system, as the T cell response is critical to clearing neuroinvasive WNV infection^28^. Although Treg frequency does increase in the secondary lymphoid organs (SLO) and brain following WNV infection^16^ the absence of MAVS within Tregs does not alter this frequency or cell number in the brain (Fig. 5c). Therefore, it appears unlikely that MAVS is required for *in vivo* Treg expansion following WNV infection, or for Treg suppressive function, at least as mediated by CTLA-4, GITR, or CD73.

### Immunity to WNV is not compromised in the absence of Tregs expressing MAVS

While none of the Treg suppressive marker expression levels examined were altered by the absence of MAVS signaling within Tregs (Fig. 5b), there are many other mechanisms by which Tregs can suppress T cell responses^29^. Therefore, we next examined the T cell response following WNV infection in the presence or absence of Tregs expressing MAVS. In contrast to full Mavs^−/−^ mice, the overall frequency of CD4 and CD8 T cells in both the spleen and brain is nearly identical in Mavs^−/−^ Treg chimeric mice as compared to WT controls, indicating that bulk T cell frequency and/or expansion is independent of MAVS expression by Tregs following WNV infection at the early visceral phase (Fig. 5d,e). In addition, we found that T cell infiltration of the CNS in the late visceral phase was no different between Mavs^−/−^ Treg chimeras and controls (Fig. 5e). When T cells were analyzed for expression of activation and functional markers such as CD44 and CXCR3, we found that both MAVS-deficient or -sufficient Treg groups were comparable (data not shown). Finally, when the WNV-specific CD8 T cell response was examined using MHC class I tetramer staining, similar frequencies of NS4b-specific CD8 T cells were measured in the spleens and brains of mice independent of Treg expression of MAVS (Fig. 5d,e). In sum, our results indicate that MAVS expression by Tregs is unlikely to be required for an effective T cell response against WNV infection *in vivo*.

## Discussion

Two major paradigms of immunology, clonal selection and pattern recognition, describe mechanistically how cells of the immune system can sense and distinguish foreign invaders that are potentially dangerous to the host. Since it has been shown that Tregs are involved in the immune responses to many distinct pathogens, it appears that this T cell subset must also be able to detect the presence of microbes in order to exert their potential modulatory effects on the ensuing immune response. We reasoned that this could be done via RLR sensing, as our previous study using mice deficient in MAVS showed a lack of Treg expansion following WNV infection, along with uncontrolled inflammation and increased mortality^13^. However, here we show that rather than a lack of expansion, we see a reduced expression of Foxp3 in Tregs from WNV infected Mavs^−/−^ mice compared to WT mice. In addition, intrinsic MAVS signaling is dispensable for proper control of WNV, and further, appears not to be required for full Treg functional capacity following infection.

While our hypothesized candidate for Treg sensing of WNV infection appears dispensable, several other possibilities exist. First, it is well established that activation of T cells is dependent upon signals through the TCR interaction with MHC and the cognate peptide antigen, as well as co-stimulatory receptors such as CD28 interacting with B7 molecules on APCs. However, the role for TCR specificity in Treg activation in the course of infection remains controversial, as Tregs are thought to have a relatively high affinity for self-antigens^30^. Foxp3+ Tregs were first demonstrated to be specific for the microbe *Leishmania major* in a study where they strongly proliferated in response to *Leishmania*-infected DCs, and further, the majority of Tregs in infected tissues were found to be microbe-specific^31^. More recently, *Mycobacterium tuberculosis* (Mtb)-specific Tregs were identified following infection, and these cells were specifically of the thymically-derived, rather than peripherally-induced, lineage^32^, thereby demonstrating that cells exiting the thymus as Tregs can be specific to microbial antigens. Therefore, it is possible that Tregs do express TCRs specific for at least a subset of microbial antigens, though this has not been formally tested in the specific context of WNV infection.

Alternatively, Tregs could sense the presence of infection through Type I IFN signaling or some other type of cytokine signal. Recently, Tregs lacking surface expression of Type I IFN receptor during LCMV infection were shown to impair the development of an effective CD8+ T cell response leading to LCMV chronicity^33^. In a murine model of colitis, it has been demonstrated that type I IFNs help to maintain Foxp3 on colonic Tregs under inflammatory conditions^34^, and so it is possible that this could occur as well in the context of infection. Because Type I IFN is a key immune effector molecule downstream of RLR recognition of WNV and related viruses^35^,^36^,^37^, it is possible that Treg detection of microbial products occurs through RIG-I indirectly via type I IFN. MAVS signaling was required for WNV-triggered DC production of type I IFN *in vitro,* though serum levels from mice infected with WNV were similar between WT and MAVS-deficient mice^13^, suggesting that there are multiple redundant pathways capable of triggering the IFN cascade upon WNV infection *in vivo*. Additionally, serum levels of cytokine may not describe IFN levels in various local microenvironments (such as SLO) where Tregs would likely receive signals to promote the response to infection, and so RIG-I-mediated production of type I IFN remains a possibility as a mechanism by which Tregs could sense the presence of WNV infection, though the cellular sources of IFN as well as the timing and location of production remain unresolved.

Through our study, we have additionally identified a cell-extrinsic role for MAVS in stable expression of Foxp3 by Tregs. In WNV-infected mice lacking MAVS on all cells, there is a dramatic increase in serum levels of several pro-inflammatory cytokines, including IL-6, which is concurrent with a loss of Foxp3 expression in a large subset of Tregs (Figs 1b and 2b). Thus, we hypothesize that the pro-inflammatory environment promoted by IL-6 and other cytokines, in addition to uncontrolled virus replication^13^, leads to a change in host strategy away from the suppressive activity of Tregs, perhaps resulting in a more Th17-type profile, as suggested by the elevation in serum IL-17 (Fig. 2b) and increased total numbers of RORγT+ CD4 T cells (Fig. 2a) that could be considered to be Th17 cells. Indeed, it has previously been shown that IL-6 coordinates with IL-1β to block the suppressive effect of Tregs on CD4+ T cells, at least in part by controlling their responsiveness to IL-2^38^. Interestingly, stimulation of Tregs in the presence of IL-6 was previously reported to result in loss of Foxp3 expression and the acquisition of a Th17 phenotype^25^,^39^, and indeed, IL-6 signaling in psoriasis prevents immune suppression by Tregs^40^. Thus, it appears that under extreme “emergency” conditions, such as during a virus infection when the host lacks a key innate immune sensor, an over-reaction may lead to conversion to a fully inflammatory response, including diminishing the Foxp3 levels in Tregs, which may subsequently result in diminished function given enough time. Importantly, although we identified no difference in Treg suppressive markers at day 6 p.i., the time at which Mavs^−/−^ mice have diminished Foxp3 expression on Tregs compared to WT mice, these mice succumb starting at day 7 post-infection, thus precluding our ability to examine any downstream effects on Treg suppressive capacity in the full knockout.

In sum, our results suggest that Tregs do not sense WNV infection through intrinsic RLR signaling. However, given the appreciated role that Tregs play in anti-WNV immunity^15^,^16^, it is increasingly vital that we understand how they sense the presence of infection and subsequently initiate their role in the immune response. A thorough characterization of how Tregs detect virus infection is critical if these cells are to be leveraged for therapeutic strategies, as it would be the key to directing these cells toward the type of immune responses required to prevent and/or eliminate infections.

## Materials and Methods

### Ethics Statement

All animal experiments were approved by the University of Washington Institutional Animal Care and Use Committee (IACUC protocol #4327–01 and 4158-01) and the Fred Hutchinson Cancer Research Center (IACUC protocol #1810). The Office of Laboratory Animal Welfare of the National Institutes of Health (NIH) has approved the University of Washington (#A3464-01) and the Fred Hutchinson Cancer Research Center (#A3226-01), and this study was carried out in strict compliance with the Public Health Service (PHS) Policy on Humane Care and Use of Laboratory Animals.

### Virus

West Nile virus TX-2002-HC (WN-TX) was generously provided by Dr. Jason Netland (University of Washington) and propagated as previously described^13^,^41^. Working stocks were generated from supernatants collected from infected Vero cell lines, and stored at −80 °C.

### Mice

*MAVS-*deficient mice^13^ were bred and maintained under specific pathogen-free conditions in the University of Washington South Lake Union animal facility. Foxp3^*DTR*^ 19 and Foxp3^*GFP*^^42^ mice (kindly provided by Dr. Alexander Rudensky, Memorial Sloan-Kettering Cancer Center) were bred under specific pathogen-free conditions onsite in the Fred Hutch animal facility. Ly5.1 *Foxp3*^*DTR*^ mixed bone marrow chimeric mice were transported to the animal facility at the University of Washington for all West Nile virus infection studies. Wild-type C57BL/6 J control mice were purchased through The Jackson Laboratory (Bar Harbor, ME USA) and housed in the University of Washington animal facility.

### 90%/10% Mixed bone marrow chimeras

One day prior to bone marrow transplant, recipient Foxp3-DTR Ly5.1 mice were irradiated (900 Rads). On the day of transplant, Foxp3-DTR Ly5.1, WT (C57BL/6) and Mavs^−/−^ mice were euthanized and femurs and tibias from both hind legs collected from each mouse. Bones were irrigated with 1X PBS and bone marrow was homogenized through 100 μM cell strainers. Red blood cells were lysed using ACK lysis buffer and bone marrow cell suspension was enumerated using trypan blue staining. Appropriate dilutions were calculated to ensure a 90% Foxp3-DTR Ly5.1 + 10% WT or Mavs^−/−^ bone marrow mixture was achieved. Cell preparations were introduced intravenously through the tail vein of recipient Foxp3-DTR Ly5.1 mice. Recipient mice were provided with antibiotic (Baytril ®, Bayer Corp) supplemented water and recovery food gel for 2 weeks post transplant. Mice were then allowed to recover for a total of 12 weeks prior to use in any experiments.

### 90%/10% Mixed bone marrow chimeras – Diphtheria toxin administration

Four to five days prior to infection with WNV, mixed bone marrow chimeras were intraperitoneally injected with 10 μg/Kg of diphtheria toxin (Calbiochem, Merck KGaA, Damrstadt, Germany). Chimeras were then bled via intra-orbital sinus puncture to ensure that either Mavs^−/−^ or WT-B6 Tregs had repopulated the Treg niche. Roughly 8–12 hours later mice were infected with WNV as described below with a continuing schedule of 5 μg/Kg intraperitoneal DT administration every other day thereafter throughout the course of infection in order to maintain ablation of endogenous DTR-expressing Tregs.

### *In vivo* Infection

Age-matched Mavs^−/−^ or mixed bone chimeras and WT controls were infected through sub-cutaneous injection of the left foot pad with 1000 PFU West Nile virus TX-2002-HC delivered in a 40 μl dilution of sterile 1X PBS.

### TGFβ, IL-6 and IL-17 ELISA

Whole blood was collected from WNV infected Mavs^−/−^ and WT control mice 6 days p.i. Blood was allowed to coagulate at room temperature for >1 hour and then centrifuged at 3000 rpm for 10 mins. Supernatant serum layer was then carefully transferred to a new tube and stored at −80 °C prior to ELISA assays. On day of ELISA, samples were allowed to thaw at 4 °C and then UV irradiated for 30 mins in BSL2+ conditions to inactivate WNV. TGFβ concentration was measured as per manufacturer’s protocol in Mouse TGF-beta1 Platinum ELISA kit (Affymetrix, eBioscience, Inc. CA, USA). Samples were diluted 1:500 as per kit instructions and spectrophotometric data collected at 1° wavelength = 450 nm and 2° wavelength = 620 nm. IL-6 concentration was measured as per protocol included in Mouse IL-6 ELISA Ready-Set-Go! ® kit (eBioscience, Inc). Samples were diluted 1:10 and analyzed at 1° wavelength = 450 nm and 2° wavelength = 570 nm. Finally, IL-17 concentration was measured as per protocol included in Mouse IL-17A (homodimer) ELISA Ready-SET-Go! ® kit (eBioscience, Inc) with overnight incubation of detection antibody with overlayed sample or standard to increase assay sensitivity. Samples were diluted 1:5 and analyzed at 1° wavelength = 450 nm and 2° wavelength = 570 nm.

### *In vitro* Treg suppression assay

Mavs^−/−^ or WT mice on a B6 background were euthanized, spleens harvested and a passed through a 100 μm cell strainer to create a single cell suspension. Cell suspension was then enriched for Tregs using a mouse CD4+CD25+ magnetic bead-based column regulatory T cell isolation kit (Cat: 130-091-041) as per manufacturer’s instructions (MACS Miltenyi Biotec, Germany). Flow through was stained with CFSE (eBioscience, Inc, San Diego, CA) and used as Tconv population. Briefly, spleens from WT mice were homogenized to form a single cell suspension, RBCs lysed, and remaining splenocytes irradiated. Irradiated splenocytes served as antigen presenting cells. Decreasing concentrations of Tconv to enriched Tregs were cultured with 5 × 10^4^-irradiated splenocytes supplemented with 1 μg/well anti-CD3 antibody. Samples were incubated for 80 hours at 37 °C and CFSE dilution was measured through flow cytometry. Detailed assay protocol is as described in Chapter 2, Section 3.2 Variations of Basic Protocol: Antigen Presenting Cell Activation^43^.

### *In vitro* Proliferation assay

Spleens were harvested from Mavs^−/−^ or WT mice and homogenized through a 100 μm cell strainer to create a single cell suspension. RBCs were lysed and Tregs isolated using the EasySep™ Mouse CD4+CD25+ Regulatory T Cell Isolation Kit (STEMCELL Technologies Inc, Vancouver, BC, Canada) as per manufacturer’s instructions. Tregs were subsequently labeled with CFSE (eBioscience, Inc, San Diego, CA). For DC isolation, spleens were collected from WT mice, minced in a cell culture well and incubated in a DMEM/collagenase D/DNAse I (Roche), media solution with mechanical agitation for 30 mins at 37 °C. The digestive solution was inactivated after incubation by resuspending the sample in HBSS/EDTA and re-incubating the minced tissue at 37 °C for 5 mins. The digested tissue was then homogenized by pushing the sample through a 100 μm cell strainer and RBCs lysed using ACK lysis buffer. Dendritic cells were then isolated using CD11c microbead magnetic column enrichment (MACS Miltenyi Biotec, Germany). Isolated DCs were incubated with heat-inactivated WNV for 1hr at 37 °C or left “naïve”. Tregs were plated at 5 × 10^4^ cells per well and co-cultured with 2 × 10^5^ DCs (either WNV loaded or not) for 3 days at 37 °C. Anti-CD3 and anti-CD28 (1:10 dilution from 1 μg/mL stock) were used as a polyclonal stimulus for positive controls. After 3 days samples were stained for viability as well as T cell and DC markers and CFSE dilution analyzed via flow cytometry.

### Flow cytometry

Mavs^−/−^ Treg and WT chimeras were infected as described above. On scheduled collection days mice were euthanized in accordance with University of Washington IACUC regulations and spleens collected. Mice were then perfused through the left ventricle of the heart with ~10 mL cold PBS. Brains were then harvested into 5 mL of RPMI culture media supplemented with 10% fetal bovine serum (FBS), Pen/Strep, L-glutamine, HEPES buffer and sodium pyruvate. Spleens were homogenized and passed through a 100 μm cell strainer to create a single cell suspension, RBCs lysed using ACK lysis buffer and cells enumerated, after trypan blue staining, using a hemocytometer. Splenocytes were then diluted to a concentration of 1 × 10^7^ cells/mL using a 1XPBS/0.5% FBS solution and 100 uL for a total of 1 × 10^6^ cells/well per sample. Brains and collection media were poured into culture wells of a 6-well culture plate and homogenized using the frosted ends of 2 microscope slides. Homogenate was then returned to collection conical and designated culture well rinsed with 600 μL of collection media. Tubes containing 5.6 mL of homogenate were then overlayed with 2.4 mL of hypertonic Percoll density centrifugation media (1:10 of 10X PBS: Percoll), (GE Healthcare Life Sciences, Pittsburgh, PA) for a final Percoll concentration of 30%. Samples were vortexed and spun at 1250 RPM for 30 mins at 4 °C. After spin, supernatant was aspirated and pellet resuspended in 2 mL 1XPBS/0.5% FBS solution and cells enumerated. Samples were resuspended in 600 μL and 100 μL plated per well. Cells were then stained (all incubation steps were at 4 °C) for viability using LIVE/DEAD® Fixable Aqua Dead Cell Stain (ThermoFisher Scientific) at 1:1000 for 30 mins. This was followed by 10 min incubation with anti-CD16/32 (clone: 93) at 1:500. Cells were then resuspended in 50 μL antibody cocktails for 15 mins. Antibody markers included CD3-BUV395 (145-2C11) CD4-BV605 (RM4-5), CD8-BV650 (53-6.7) and Foxp3-Alexa700 (FJK-16s). WNV NS4b-H2D^b^ tetramer-APC (generated by the Immune Monitoring Lab, Fred Hutch tetramer core) was used to determine frequency of WNV-specific CD8+ T cells. Additional antibodies for Treg characterization included: CD73-V450 (ebioTy/11.8), GITR-PECy7 (DTA-1), CTLA-4-APC (UC10-4B9). Cells were fixed and permeabilized for 30 mins before intracellular staining antibodies were applied. Data was collected via flow cytometry using a BD LSRII and BD FACSDiva Software. Sample analysis was performed using FlowJo software.

### RNA Extraction

After CO_2_ euthanasia, mice were perfused through the left ventricle of the heart with ~10 mL cold PBS. The brain cavity was accessed and the cerebellum removed and immediately placed into pre-weighed tubes containing 5 mL RNA*later* Stabilization Reagent (Qiagen, Valencia, CA). Samples were reweighed to determine weight of cerebellum alone and placed in −80 °C freezer. Brains were thawed at 4 °C, homogenized using a handheld homogenizer and total RNA extracted following protocol instructions included with the RNeasy® Lipid Tissue Mini Kit (Qiagen, Valencia, CA). RNA was eluted into RNase-free 10 mM TE Buffer, pH 7.0 and RNA concentration measured using a Nanodrop 2000 UV-Vis spectrophotometer (Thermo Scientific, Waltham, MA).

### WNV DNA standard

Competent E. coli cells transformed with kanamycin resistant plasmids containing the WNV PCR target region were grown in kanamycin-containing media and plasmids isolated using the Qiagen Plasmid Mini kit (Qiagen, Valencia, CA). The plasmid concentration was determined using a Nanodrop 2000 UV-Vis spectrophotometer (ThermoScientific) and 10 fold standard dilution series was generated spanning a concentration of 1e10 to 1e2 plasmids/5ul.

### qRT-PCR for WNV RNA

WNV primers and probe were derived as previously described^44^. The fluorogenic probe was synthesized with a 5′ reporter dye 6-carboxyfluorescien (6-FAM) and a 3′ quencher dye 6-carboxytetramethylrhodamine (5′-TAMRA). Primers and probe were generated as custom assays from Integrated DNA Technologies (IDTDNA, Coralville, Iowa). qRT-PCR assays were performed using the SuperScript® III Platinum® One-Step Quantitative RT-PCR System (Life Technologies, Grand Island, NY). Reactions were carried out in a total volume of 20 uL, containing 5 uL of template RNA, 1X reaction mix, 500 nM final concentration for forward and reverse primers and 250 nM final concentration for probe, 0.4ul ROX dye, 0.4 uL RT/Taq enzyme mix and brought up with nuclease free water. After adding the reaction mixture and template RNA to MicroAmp® Fast Optical 96-Well Reaction Plates (Applied Biosystems, Inc., Foster City CA), reverse-transcription and amplification were carried out on the ABI 7900HT Fast Real-Time PCR System (Applied Biosystems, Inc., Foster City CA) in standard mode. Cycling conditions were as follows: 50 °C for 15 minutes hold (cDNA synthesis step), 95 °C for 2 minutes hold, 40 cycles of 95 °C for 15 seconds followed by 60 °C, 1 minute.

## Additional Information

**How to cite this article**: Da Costa, A. *et al*. Extrinsic MAVS signaling is critical for Treg maintenance of Foxp3 expression following acute flavivirus infection. *Sci. Rep.* **7**, 40720; doi: 10.1038/srep40720 (2017).

**Publisher's note:** Springer Nature remains neutral with regard to jurisdictional claims in published maps and institutional affiliations.
